# Sequential Changes in Antioxidant Potential of Oakleaf Lettuce Seedlings Caused by Nano-TiO_2_ Treatment

**DOI:** 10.3390/nano11051171

**Published:** 2021-04-29

**Authors:** Rita Jurkow, Andrzej Kalisz, Dalibor Húska, Agnieszka Sękara, Soheila Dastborhan

**Affiliations:** 1 Department of Horticulture, University of Agriculture in Krakow, 29 Listopada 54, 31-425 Kraków, Poland; r.jurkow@gmail.com (R.J.); agnieszka.sekara@urk.edu.pl (A.S.); 2 Laboratory of Plant Metabolomics and Epigenetics, Department of Chemistry and Biochemistry, Mendel University in Brno, Zemedelska 1, 61300 Brno, Czech Republic; dalibor.huska@mendelu.cz; 3 Department of Plant Eco-Physiology, Faculty of Agriculture, University of Tabriz, Tabriz 5166616471, Iran; dastborhan.s@gmail.com

**Keywords:** antioxidants, *Lactuca sativa* var. *foliosa*, nanoparticles, sequential sampling, titanium dioxide

## Abstract

Nowadays, there is an increasing interest in nanoparticle (NP) technology used in household and industrial products. It could cause an accumulation and dispersion of NPs in the environment, with possible harmful effects on living organisms. Nanoparticles significantly affect plants and alter their physiology and biochemical pathways, and nanotechnology can be used to improve plant characteristics that are desirable by humans. Therefore, more extensive studies of NP interactions with plants are still needed. The aim of this report is to investigate the effect of TiO_2_ nanoparticles (TiO_2_-NPs) on the enzymatic and non-enzymatic antioxidants, fresh and dry weights, and malondialdehyde contents in oakleaf lettuce seedlings. Plants were foliar treated with a 0.75% suspension of TiO_2_-NPs, while control plants were sprayed with deionized water. Leaves were sampled 4, 7, 9, 11, and 13 days after the treatment. The effects of TiO_2_-NPs were time-dependent, but the most spectacular changes were observed 4 days after the treatment. Exposure of the plants to TiO_2_-NPs significantly increased the contents of glutathione at all sampling points, total phenolics at days 4 and 13, and L-ascorbic acid at 4, 7, and 11 days after the treatment. Elevated levels of ascorbate peroxidase and guaiacol peroxidase activities were recorded at days 4 and 13, respectively. Total antioxidant capacity increased initially in treated seedlings, when compared with the control, and then decreased. On day 7, higher fresh and dry weights, as well as malondialdehyde contents in TiO_2_-NPs treated plants were observed, compared with the control. The study demonstrated that the activation of some antioxidant system components due to TiO_2_-NPs treatment was connected with the induction of mild oxidative stress, with no external symptoms of NP toxicity in oakleaf lettuce.

## 1. Introduction

Nanoparticles (NPs) are defined as aggregates of atoms or molecules with dimensions ranging from 1 nm to 100 nm, taking spherical, fibrous, or layered forms [1,2]. Nanoparticles are a natural component of the environment, although nowadays more and more engineered NPs are generated by human activity [3]. Engineered nanoparticles show physicochemical properties that may differ from their native counterparts in many aspects [4]. The growing application of nanotechnology increases the release of NPs into the environment. Several reports concern the impact of nanoparticles on plants, but still the influence of NPs on the response mechanism of living organisms is not well understood [5,6]. To date, several reports contain results showing the positive or negative effects of NPs on plants [4,5,6,7]. NPs affect seed germination, plant growth, cell wall and lipid membranes, and photosynthesis and gas exchange, and may induce oxidative stress and activate antioxidant systems [6,8]. NPs are able to generate, directly or indirectly, an excess of reactive oxygen species (ROS), with the potential to affect proteins, lipids, carbohydrates, and DNA in plants [9]. Oxidative stress causes alteration in the activity of antioxidant enzymes, inter alia, superoxide dismutase (SOD), catalase (CAT), and different peroxidases (i.e., ascorbate peroxidase APX, glutathione peroxidase GPX, and guaiacol peroxidase GPOX), and affects the concentration of several non-enzymatic antioxidants (i.e., ascorbic acid, reduced glutathione, α-tocopherol, carotenoids, and phenolics) in plants [9,10,11,12]. After the contact of NPs with plants, their physical and chemical properties are responsible for the mechanism of adsorption, uptake, transport, and biotransformation, which in turn influence phytotoxicity [9]. In this regard, it is important to carefully choose the concentration, form, and route of delivery of the NPs and the plant species [5,9]. All of these factors affect NPs–plant interactions and the intensity of plant responses.

Titanium dioxide nanoparticles (TiO_2_-NPs) are one of the most widely produced NPs in the world [13]. TiO_2_-NPs have become a common additive in paints, plastics, and personal care products (cosmetics, sunscreens), as well as in food—as the additive E171—and in medical applications [14]. In agriculture, TiO_2_-NPs are considered as beneficial elements for plant growth and development [15]. Low concentrations of titanium dioxide NPs have been shown to improve crop performance by the better growth of roots or leaves; they stimulate the activity of certain enzymes, increase the content of chlorophyll and photosynthesis activity, and also result in a better absorption of nutrients [8,16]. Li et al. [17] found TiO_2_-NPs to be nontoxic for plants, aside from having characteristics which improve plant performance. They showed that TiO_2_-NPs increased the physiological efficiency of the photosynthetic apparatus, plant growth, and yield of rapeseed. The research by Zheng et al. [18] showed an acceleration of spinach growth as well as an increase in chlorophyll synthesis and in the activity of several enzymes when TiO_2_-NPs were applied to seeds or when plant leaves were sprayed with a suspension of these NPs. Gohari et al. [19] observed the promoted growth and amelioration of salinity stress in the Moldavian balm treated with TiO_2_-NPs. However, some studies highlighted that plants could be adversely affected by TiO_2_-NPs [20]. At low concentrations, TiO_2_-NPs accelerated the growth of fine duckweed (*Lemna minor*), while at its high concentrations, the growth was inhibited [21]. Frazier et al. [22] observed the dose-dependent reduction of germination, plant growth, biomass, and root length of tobacco after TiO_2_-NPs treatment. As we can see, TiO_2_-NPs exhibit a dual nature of both beneficial and toxic constituents, depending upon many experimental factors [20].

Lettuce is one of the most common edible leafy vegetables in human consumption, with a promising phytochemical profile [23,24]. Although butterhead and crisphead lettuce types are the most popular on the market, the demand for green and red oakleaf lettuces has considerably increased in recent years [25]. Oakleaf lettuces are fast growing plants with delicate leaves of large leaf surface, making the plant a good model for studies with foliar applied NPs. It is worth noting that lettuce is often used in studies assessing changes in plant metabolic pathways under the influence of abiotic stress, including the effects of NPs on these plants [26,27,28,29].

In this study, the effects of the foliar application of TiO_2_-NPs on the physiological responses of oakleaf lettuce seedlings, covering antioxidant enzyme activities and non-enzymatic antioxidant contents, as well as on fresh and dry weights of the plants, were investigated. We chose a low dose of TiO_2_-NPs, based on our previous research [30], but we also focused on a time-dependent response of plants to NPs treatment. This allows us to better track changes in plant metabolism and assess the severity of stress that could be caused by TiO_2_-NPs, the activity of antioxidant mechanisms, and the process of plant acclimatization.

## 2. Materials and Methods

Seedlings of oakleaf lettuce cv. ‘Kiribati’ (Rijk Zwaan) were obtained from Grupa Producentów Rozsad Krasoń Sp. z o. o. (Piaski, Poland). Seeds were sown in cubic peat pots (64 mL) placed in plastic boxes (each contained 150 cubes). Biofertilizer Goëmar Goteo (Arysta LifeScience Polska Sp z o. o., Warsaw, Poland), which contained 3% P_2_O_5_ and 5% K_2_O, and *Ascophyllum nodosum* filtrate was used at the two-leaf stage at a concentration of 0.3%. No other additional fertilization was applied during trial. The main experiments were carried out in the greenhouse of the University of Agriculture in Krakow, Poland. Two-week-old plants having 4–5 leaves were placed on a flooded table. Conditions in the greenhouse were set at 23/19 °C day/night temperature, ca. 75% relative humidity, and seedlings were exposed to natural photoperiod (ca. 16 h) and daylight. Irrigation was carried out by flooding the table as needed, always up to ¾ height of the cubic pots, and no sprinkler irrigation was used.

The titanium (IV) dioxide nanoparticles (TiO_2_-NPs), anatase form, were purchased from PlasmaChem GmbH (Berlin, Germany), as 5 wt% aqueous colloidal suspension. The average particle size of TiO_2_-NPs was 6 ± 2 nm, and their specific surface area was ca. 140 m^2^·g^−1^. Nanoparticles of TiO_2_ were applied once, on the plant leaves, with the concentration of 0.75 wt%. The volume of the suspension per seedling box was 50 mL. Control plants were sprayed with deionized water at the same time. The leaves were sampled five times: 4, 7, 9, 11, and 13 days (t1, …, t5, respectively) after the treatment. In the experiment, 1500 seedlings were used, half as controls, half as nanoparticle treated plants. For each sampling and treatment, 150 plants were taken from the seedling boxes (50 plants per replicate). The leaves were washed with tap water and rinsed with deionized water. The leaves from each particular treatment and replicate were mixed, and fresh laboratory samples were made to determine fresh weight, dry weight, L-ascorbic acid, and glutathione. The rest of the leaves were inserted into an ultra-deep freezer to a temperature of −40 °C for further chemical analyses.

The malondialdehyde (MDA) content was determined using the method described by Dhindsa and Matowe [31]. The leaves (1.5 g) were homogenized with 6 mL of 0.1% trichloroacetic acid (TCA) solution. The extract was centrifuged at 13,968× *g*, for 15 min, at 18 °C. The supernatant (0.5 mL) was collected and mixed with 1 mL 20% TCA, containing 0.5% thiobarbituric acid and 0.1 M K-phosphate buffer (pH 7.6). The absorbance of the supernatant was read at 532 and 600 nm (UV-VIS Helios Beta spectrophotometer, Waltham, MA, USA). An extinction coefficient of 155 mM^−1^·cm^−1^ was used for calculating MDA contents.

The total antioxidant activity was monitored according to the method described by Molyneux [32], using the DPPH radical (2,2-diphenyl-1-picrylhydrazyl). For the extraction, 2.5 g of each of the samples were weighed out, grounded with 10 mL 80% methanol, and centrifuged (3492× *g*, 10 min, 4 °C). The supernatant (0.1 mL) was mixed with 4.9 mL of 0.1 mM DPPH^•^ dissolved in 80% methanol. The reaction mixture was shaken and incubated in the dark, at room temperature, for 15 min. Absorbance was measured at 517 nm against the blank using UV/VIS Helios Beta spectrophotometer. The antioxidant activity was calculated as DPPH [%] = [(A0 − A1) / A0] × 100, where A0 and A1 are the absorbance of the reference and test solutions, respectively.

The reduced form of glutathione (GSH) was assayed using the method described by Guri [33], with some modifications. In the GSH assay, 2.5 g of fresh leaves were chopped and homogenized with 6.0 mL 0.5 mM EDTA and 3% trichloroacetic acid (TCA) in an ice bath (4 °C). The extract was centrifuged at 13,968× *g*, for 10 min, at 4 °C. Supernatant (2 mL) was mixed with 5 mL K-phosphate buffer (pH = 7.0) to bring solution pH to the value of c.a. 7.0. From this mixture, 2 mL was taken into the tube where, again, 1 mL K-phosphate buffer was added. Ellman’s reagent (5,5-dithiobis-2-nitrobenzoic acid) in the amount of 0.1 mL was also added to the sample. The content of reduced glutathione was assessed by measuring absorbance at 412 nm on UV-VIS Helios Beta spectrophotometer, against a blind sample, prepared as described above, but without Ellman’s reagent.

L-ascorbic acid was determined by Tillman’s titration method [34]. The plant material (50 g) was homogenized with 200 mL acetic acid as acidity regulator, and, after 30 min, the extract was titrated with Tillman’s reagent (2,6-dichlorophenolindophenol). Excessive dye in an acidic environment gives a pink color and marks the end point of the titration. The content of L-ascorbic acid in the sample was calculated based on the amount of Tillman’s reagent used for titration.

Phenolic compounds were determined according to the Folin–Ciocalteu method, as described by Djeridane et al. [35]. Briefly, the plant material (2.0 g) was mixed with 10 mL of 80% methanol, and, after that, centrifuged (3492× *g* for 10 min at 4 °C). The plant extracts (0.1 mL) were mixed with 2 mL of sodium carbonate. After 2 min, Folin–Ciocalteu’s reagent (0.1 mL), mixed with deionized water (1:1 *v*/*v*), was added to the test tubes. The mixture was incubated at room temperature (22 °C) in the dark, for 45 min. The absorbance was measured at 750 nm using the UV-VIS Helios Beta spectrophotometer. The total phenolics content was calculated on the basis of the calibration curve of gallic acid and expressed as gallic acid equivalents (GAE) per 1 g fresh weight (FW).

Peroxidase activity was assayed as follows: leaf samples (2 g) were homogenized in an ice bath (4 °C) in 10 mL 50 mM potassium phosphate buffer (pH 7.0), containing 1 mM ethylene diamine tetraacetic acid (EDTA), 1% soluble polyvinyl pyrrolidone (PVP), and 1 mM phenylmethylsulfonyl fluoride (PMSF). Subsequently, the mixture was centrifuged at 13,968× *g*, for 15 min, at 4 °C. The ascorbate peroxidase (APX, EC 1.11.1.11) assay procedure was conducted according to the method of Nakano and Asada [36]. The activity of APX was determined in 50 mM potassium phosphate buffer (pH 7.0), containing 0.5 mM ascorbate (AsA) and 0.1 mM hydrogen peroxide in a total volume of 0.15 mL. The enzymatic reaction was started by adding 0.2 mL of enzyme extract to the mixture. The hydrogen peroxide-dependent oxidation of AsA was followed by monitoring the decrease in absorbance at 290 nm (Helios Beta spectrophotometer), assuming an absorption coefficient of 2.8 mM^−1^·cm^−1^. The APX activity was presented as µg AsA·min^−1^·g^−1^ FW. The activity of guaiacol peroxidase (GPOX, EC 1.11.1.7) was determined by the use of guaiacol as a substrate [37]. The reaction medium contained 50 mM phosphate buffer (pH 7.0), along with guaiacol (4%) and H_2_O_2_ (1%). The enzymatic reaction was started by adding 0.4 mL of enzyme extract to the mixture. The absorbance of supernatant was measured at 470 nm (Helios Beta spectrophotometer) using the molar extinction coefficient 26.6 mM^−1^·cm^−1^. The GPOX activity was presented as μmol tetraguaiacol·min^−1^·g^−1^ FW.

Seedling shoots (15 plants per replicate) were weighed with a Sartorius A120S balance (Sartorius AG, Göttingen, Germany) to assess fresh weight (FW). The determination of dry weight was performed by the dryer method [38], using the same plants as for FW. The fragmented samples were weighed, using Sartorius A120S analytical balance, and dried at 65 °C, for 48 h. After drying, the samples were weighed again and the results were converted into the percentage of dry weight in fresh weight, and then re-calculated into dry weight per shoot.

The total content of titanium (Ti) was performed as follows: the leaves were thoroughly washed with tap water and rinsed with deionized water, and, after that, the plant samples were oven-dried at 65 °C, to a constant weight, and ground to a fine powder, using a Pulverisette 14 ball mill (Fritsch GmbH, Idar-Oberstein, Germany) and a 0.5 mm sieve. A Mars 5 Xpress microwave digestion system (CEM Corporation, Matthews, NC, USA) and 100 mL TFM vessels were used for microwave mineralization of the samples. The samples (3 g) were placed into the particular vessel and mineralized at 200 °C in 10 mL of 65% super-pure HNO_3_ (Merck no. 100443.2500), the method of which was described in detail in a previous report [39]. Samples, after cooling to room temperature, were transferred to 25 mL flasks with redistilled water to the final volume of 25 mL. The titanium content was analyzed using an iCAP TQ ICP-MS/MS triple-quadrupole spectrometer (ThermoFisher Scientific, Bremen, Germany). Determination was carried out using the S-SQ-KED measurement mode for ^49^Ti isotope.

The software Statistica 13.3 (TIBCO Software Inc., Palo Alto, CA, USA) was used for statistical calculations. The analysis of variance ANOVA (two-way) was used to analyze the obtained results. Means (*n* = 3) were gathered into homogeneous groups by using the Fisher’s test, with the significance level of *p* ≤ 0.05. The effects of nanoparticles (NPs), sampling time (tn), and the interaction NPs × tn were evaluated at three levels of significance: *p* ≤ 0.05 (*), *p* ≤ 0.01 (**), and *p* ≤ 0.001 (***). The results were expressed as mean ± SD (standard deviation).

## 3. Results and Discussion

**Figure 1:**
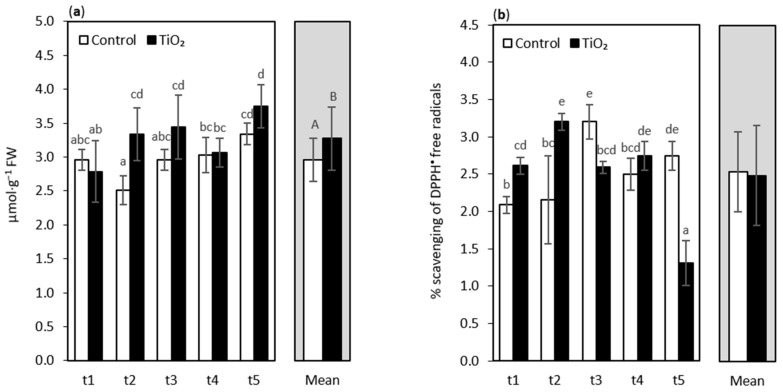
Malondialdehyde (MDA) content (**a**) and total antioxidant capacity (TAC) expressed as % scavenging of DPPH^•^ free radicals (**b**) of oakleaf lettuce seedlings treated with TiO_2_-NPs applied on the leaves as a 0.75% suspension, while the control plants were sprayed with deionized water. Plants were sampled at 4, 7, 9, 11, and 13 days after treatment (sampling time: t1, …, t5, respectively). Lower-case letters above bars highlight the significant differences for interaction effects, and capital letters indicate the differences between the means for the treatment groups (NPs treated plants and control plants), according to Fisher’s test (*p* ≤ 0.05). Missing letters indicate no significant differences. Error bars represent standard deviations (±SD).

Excessive ROS level causes in plants the peroxidation of lipids in biological membranes, and malondialdehyde (MDA) content has long been used as a lipid peroxidation marker in studies related to oxidative stress and redox signaling [40]. In the present study, after the application of TiO_2_-NPs as a 0.75% suspension to oakleaf lettuce, the level of MDA was statistically similar to control plants at most sampling points, although some slight increases were noted at t3–t5 (Figure 1a). A significant increase in MDA levels was observed only in day 7 (t2) of the treatment, by ca. 33%, in comparison with the control seedlings. The mean values for the treatments showed a generally higher content of MDA in TiO_2_-NPs treated plants, when compared with the control. The higher content of MDA, especially observed at the t2 sampling point, is in agreement with the results from other reports, for example, for the Moldavian balm (*Dracocephalum moldavica*), where high doses of TiO_2_-NPs (10 and 20 ppm) increased the content of MDA linearly, under normal irrigation conditions [41]. On the other hand, Latef et al. [42] did not observe any significant change in the MDA levels in broad bean plants treated with 0.01–0.03% TiO_2_-NPs, as compared with the control plants grown under not-saline conditions, even though the tested concentrations of NPs were very low in this case. In our opinion, the harmful influence of MDA on cell membranes, despite the observed impact, especially at t2, has been effectively limited by the activated antioxidant system, as can be seen in following parts of the report.

Total antioxidant capacity (TAC) increased significantly in the seedlings in day 4 (t1) and day 7 (t2) of TiO_2_-NPs treatment (Figure 1b). Thus, a direct response of the plants in this respect to the NPs treatment was observed. The increase in TAC in the TiO_2_-NPs plants reached 25% and 48%, respectively, for t1 and t2 samplings, compared with the control. Kőrösi et al. [43] showed that the ferric-reducing antioxidant power (FRAP) and Trolox equivalent antioxidant capacity (TEAC) values were significantly influenced by TiO_2_-NPs treatment (1 mg·mL^−1^) of *Vitis vinifera* leaves—almost all tested grapevine cultivars enhanced the level of antioxidant activity. Additionally, Ghorbanpour [44] described (for *Salvia officinalis*) that the application of TiO_2_-NPs significantly improved the antioxidant activity of the plant extracts compared with the control. However, in our study, the ability to scavenge DPPH^•^ decreased significantly at t3 and especially at t5 sampling points, while TAC on day 11 after the treatment (t4) was statistically similar for treated and non-treated seedlings. The highest decrease in the ability to scavenge DPPH radicals occurred in lettuce 13 days after NPs treatment (t5), as it was 52% lower than in non-treated seedlings. As we know, DPPH radical scavenging activity is a measure of non-enzymatic antioxidant activity [45]. In the present study, higher levels of scavenging activity in seedlings subjected to TiO_2_-NPs at t1 and t2 sampling usually corresponded with higher glutathione and L-ascorbic acid content, and partially with total phenolics level in the plants sampled 4 days (t1) after NPs treatment. The decrease in DPPH radical scavenging activity of the plants at the t5 sampling point, due to TiO_2_-NPs application, was much more noticeable than in phenolics, which also decreased at that time.

**Figure 2:**
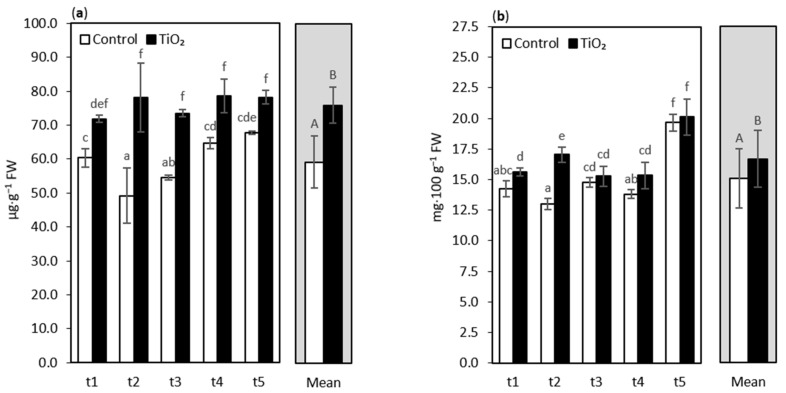
Glutathione (GSH) content (**a**) and L-ascorbic acid (AsA) content (**b**) of oakleaf lettuce seedlings treated with TiO_2_-NPs applied on the leaves as a 0.75% suspension, while the control plants were sprayed with deionized water. Plants were sampled 4, 7, 9, 11, and 13 days after treatment (sampling time: t1, …, t5, respectively). Lower-case letters above bars highlight significant differences for interaction effects, and capital letters indicate the differences between the means for the treatment groups (NPs treated plants and control plants), according to Fisher’s test (*p* ≤ 0.05). Error bars represent standard deviations (±SD).

Glutathione (GSH) is a water-soluble antioxidant that plays an essential role in scavenging non-radicals such as ^1^O_2_ and H_2_O_2_, and free radicals such as ^•^OH and O_2_^•−^ [10,19,46]. Moreover, glutathione protects the thiol-groups of enzymes located in the chloroplast stroma and participates in the production of α-tocopherol and ascorbate [47]. The application of TiO_2_-NPs on oakleaf lettuce seedlings caused a significant rise in glutathione, which was observed in all sampling points (t1–t5) compared with the control (Figure 2a). The highest increase in glutathione levels (by ca. 59%) was noted 7 days after NPs treatment. The significant effect of TiO_2_-NPs treatment on GSH was also confirmed by averaged values for main effects. It was reported that CeO_2_-NPs and In_2_O_3_-NPs caused higher GSH biosynthesis in *Arabidopsis thaliana* [48]. Larue et al. [26] described that TiO_2_-NPs treated lettuce plants contained almost twice the amount of GSH, compared with the control plants. Undoubtedly, glutathione enhances plant tolerance to different abiotic stresses, including stress caused by metals [49]. GSH is a key component in metal ions scavenging due to the high affinity of metals to its thiol (-SH) group and as a precursor of phytochelatins (PCs) [50,51]. Detoxification is done by metal conjugation with the thiol group in GSH and through the transport of such molecules to the vacuole [52]. Plant cells also synthesize different metabolites that bind the metal ions to decrease their free cytosolic concentration [53]. Phytochelatins are an example of such compounds that form the metal-PCs complexes which are then transported and sequestered in vacuoles of plant cells [49,52]. Metals in the cytosol may be neutralized by active efflux, not only to the vacuole, but also to the apoplast by specific transporters [53]. As previously mentioned, GSH plays an important role in the tackling of ROS generated in plants subjected to NPs (or other stressors), because the cysteine residue on GSH renders it an important antioxidant, which, in addition to its primary antioxidant capacities, acts as a substrate for the regeneration of other essential antioxidants [51].

Ascorbic acid (AsA) is one of the universal non-enzymatic antioxidants, having a substantial potential of scavenging ROS; moreover, it is a major component of the ascorbate-glutathione (AsA-GSH) cycle, and it helps to modulate oxidative stress in plants by controlling ROS detoxification, both alone and in co-operation with glutathione [54]. In our study, significantly higher contents of L-ascorbic acid of TiO_2_-NPs treated plants, in comparison with the control, were observed at t1, t2, and t4 sampling points (i.e., 4, 7, and 11 days after treatment, Figure 2b). A statistical analysis of the main effect means confirmed the elevated content of L-ascorbic acid in NPs treated plants. In a previous study, where higher concentrations of TiO_2_-NPs were applied (1.5%; 3%; 6%) to the oakleaf lettuce plants, we also observed an elevated level of L-ascorbic acid when compared with the control [30]. Similarly, Silva et al. [55] found (for wheat) an increase in the levels of this compound for all TiO_2_-NP treatments (5, 50, and 150 mg·L^−1^). It must be highlighted that AsA acts as an important barrier of defense against reactive oxygen species overproduced by stress conditions, including the stress caused by NPs.

**Figure 3:**
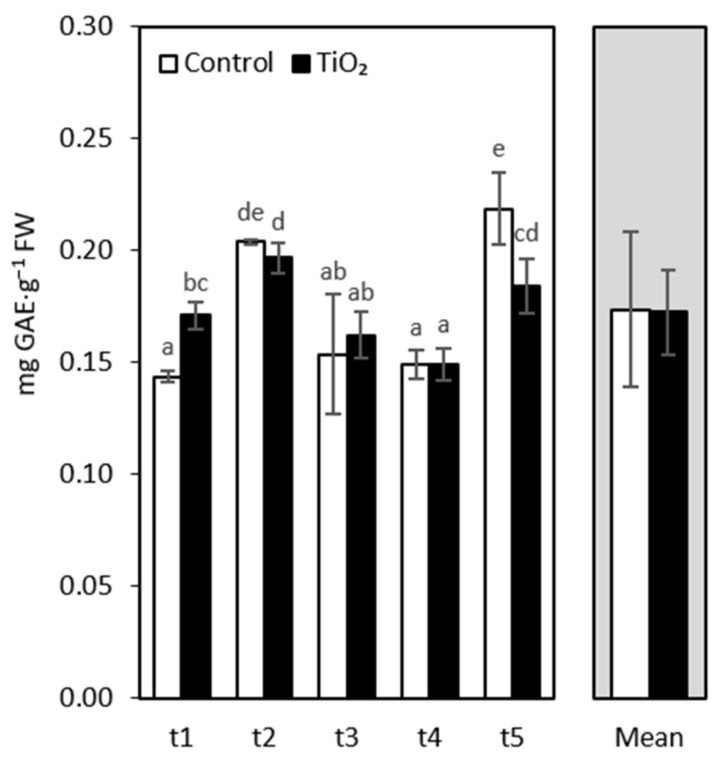
Total phenolics content of oakleaf lettuce seedlings treated with TiO_2_-NPs applied on the leaves as a 0.75% suspension, while control plants were sprayed with deionized water. Plants were sampled 4, 7, 9, 11, and 13 days after treatment (sampling time: t1, …, t5, respectively). Lower-case letters above bars highlight significant differences for interaction effects, according to Fisher’s test (*p* ≤ 0.05). Missing letters indicate no significant differences. Error bars represent standard deviations (±SD).

Phenolics have gained significant attention because of their high bioactivity and protective role in plants against oxidative stress [56,57]. In our study, we observed a strong increase in the total phenolics levels in oakleaf lettuce subjected to TiO_2_-NPs after 4 days, in comparison with the control (Figure 3). This result corresponds to the data provided by Ghorbanpour [44], who noted that sage treated with TiO_2_-NPs at certain concentrations had higher phenolic contents than non-treated plants. In addition, Comotto et al. [58] showed that phenolic concentrations of two microalgae, *Haematococcus pluvialis* and *Arthrospira platensis*, increased in the presence of TiO_2_-NPs. However, the phenolics level was not statistically affected in *Chlorella vulgaris*. Our results on phenolics, again, indicate an oxidative stress in the first days after TiO_2_-NPs treatment and the activation of antioxidant mechanisms by plants. There were no significant differences in the phenolics content between treated and non-treated lettuce, between the t2 and t4 sampling points. After 13 days (t5) from the application of NPs, the level of phenolic compounds in the control plants was higher than in those subjected to NPs. This observation corresponds to an overall decrease in the total antioxidant activity (Figure 1b).

**Figure 4:**
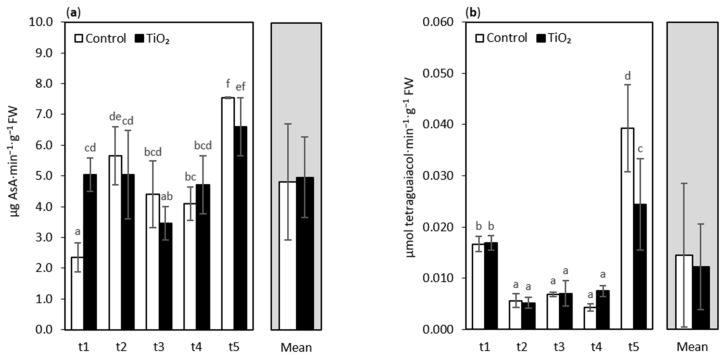
Ascorbate peroxidase (APX) activity (**a**) and guaiacol peroxidase (GPOX) activity (**b**) of oakleaf lettuce seedlings treated with TiO_2_-NPs applied on the leaves as a 0.75% suspension, while control plants were sprayed with deionized water. Plants were sampled 4, 7, 9, 11, and 13 days after treatment (sampling time: t1, …, t5, respectively). Lower-case letters above bars highlight significant differences for interaction effects, according to Fisher’s test (*p* ≤ 0.05). Missing letters indicate no significant differences. Error bars represent standard deviations (±SD).

In addition to non-enzymatic components, the antioxidant defense systems of plants contain different enzymatic antioxidants that are involved in the catalytic transformation of reactive oxygen species (ROS) and their by-products into stable nontoxic molecules [12]. Ascorbate peroxidase (APX) is a crucial enzyme regulating ROS levels, mainly H_2_O_2_, via a detoxification system called ascorbate-glutathione cycle acting in different subcellular compartments [59]. APX utilizes AsA as a specific electron donor in order to reduce H_2_O_2_ to water. In the present study, the response of lettuce seedlings to TiO_2_-NPs treatment in APX activity indeed was observed, only at 4 days after treatment (Figure 4a). APX in NPs treated plants had the lowest activity at t3 (9 days after treatment), especially when compared with much higher values at day 13 (t5). A statistical analysis of the main effects generally did not show a significant influence of NPs treatment on APX. Thus, we can conclude that APX activity increased due to plant exposure to TiO_2_-NPs, but in the case of the applied TiO_2_-NPs concentration, only immediately after application. Rao and Shekhawat [60] used copper oxide and titanium dioxide NPs on *Brassica juncea* with different concentrations (200–1500 mg·L^−1^). They observed a treatment-dependent increment in APX activity in the roots and shoots of the plant, under the influence of both the nanomaterials; however, the extent of the increase in APX was higher in the copper oxide NPs treatment, compared with titanium dioxide NPs. Lei et al. [61] reported a higher APX activity in spinach when the plants were treated with TiO_2_-NPs. In an earlier experiment on oakleaf lettuce subjected to 1.5% and 3% suspensions of TiO_2_-NPs, we showed a significant decrease in APX activity, by 25.8% and 29.0%, respectively, in comparison with the control [30]. Such a decrease was also noted in the present experiment at the t2, t3, and t5 sampling points. However, the differences in comparison with the control plants were non-significant.

Another component of the antioxidant defense system of plants is guaiacol peroxidase (GPOX), which preferably oxidizes aromatic electron donors such as guaiacol and pyrogallol at the expense of H_2_O_2_ [56]. In the present paper, we did not observe significant differences in GPOX activity between the control seedlings and the plants treated with TiO_2_-NPs. The only exception was the t5 sampling point (13 days after treatment), when a significantly higher GPOX activity was observed in the control lettuce (by ca. 38%), compared with the NPs treated plants. This contradicts the data received by Lei et al. [61], who showed an increased activity of guaiacol peroxidase in spinach subjected to TiO_2_-NPs. In a previous report, there was no effect of spraying oakleaf lettuce with 1.5% and 3% TiO_2_-NPs on the alteration of GPOX activity, but the application of TiO_2_-NPs as 6% suspension caused a significant increase in the activity of this enzyme, when compared with the control [30]. Such a decrease in GPOX activity in TiO_2_-NPs plants at the t5 sampling in the present study is difficult to explain.

**Figure 5:**
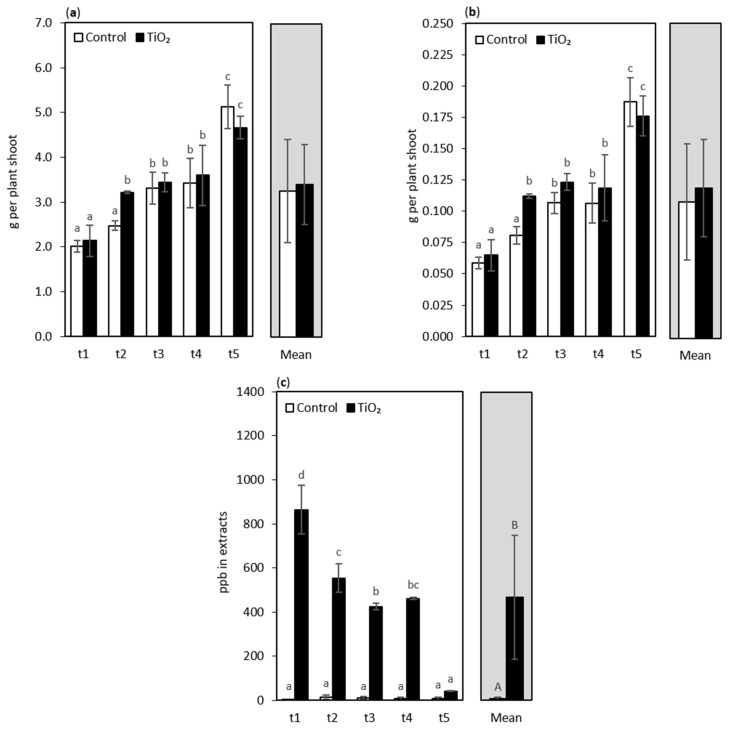
Fresh weight (**a**), dry weight (**b**), and Ti content (**c**) of oakleaf lettuce seedlings treated with TiO_2_-NPs applied on the leaves as a 0.75% suspension, while control plants were sprayed with deionized water. Plants were sampled 4, 7, 9, 11, and 13 days after treatment (sampling time: t1, …, t5, respectively). Lower-case letters above bars highlight significant differences for interaction effects, and capital letters indicate differences between the means for the treatment groups (NPs treated plants and control plants), according to Fisher’s test (*p* ≤ 0.05). Missing letters indicate no significant differences. Error bars represent standard deviations (±SD).

Various researchers reported that the TiO_2_-NPs might cause positive effects on plant growth, e.g., in canola (*Brassica napus*) [62], tomato (*Solanum lycopersicum*) [63], and Moldavian balm (*Dracocephalum moldavica*) [19]. TiO_2_-NPs stimulated plant growth usually when low concentrations were used. However, at high concentrations, these NPs did not affect or even inhibit the growth. In this report, the application of 0.75% TiO_2_-NPs increased the fresh weight (FW) of the lettuce shoot as well as its dry weight (DW) content, but only at t2 sampling (at 7 days after NPs treatment) (Figure 5a,b). There were no significant differences between NPs treated and non-treated plants in the other sampling points, at t1, t3 and t4, where, usually, FW and DW were slightly higher in the seedlings subjected to NPs. The opposite trend was observed at t5, when the control lettuce had slightly more FW and DW than TiO_2_-NPs treated plants, but this was not confirmed statistically. Summing up, the influence of TiO_2_-NPs on oakleaf lettuce growth turned out to be relatively small, as in an earlier experiment, with higher concentrations of TiO_2_-NPs (1.5–6%), no significant changes occurred in FW and DW, in comparison with the control seedlings [30].

It was expected that the application of TiO_2_-NPs on oakleaf lettuce seedlings would cause an increase in the content of Ti in plant tissues (Figure 5c). Indeed, the seedlings treated with TiO_2_-NPs had the highest content of Ti at t1–t4 sampling points (i.e., from 4 to 11 days after treatment), compared with the control plants, but the content of Ti in extracts at the t5 sampling point (13 days after treatment) was statistically similar between the treated and non-treated plants. The content of Ti in the control seedlings remained at a similar level during the experiment. The gradual decrease in Ti levels in the extracts of NPs treated plants over time should be explained by the dilution effect, due to the increasing plant biomass. Ti, applied once at the beginning of the experiment, was distributed over the growing plant tissues. The lowering content of Ti in the extracts was accompanied with stable and high glutathione content in leaf tissues, which may suggest that GSH is involved in the process of active efflux of metal ions.

An ANOVA analysis showed that the interaction between nanoparticles and the sampling time significantly influenced all tested plant parameters (Table 1). The interaction effects were significant at the level *p* ≤ 0.001. The sampling time also strongly influenced the investigated plant traits, at *p* ≤ 0.001, with the exception of malondialdehyde and glutathione content (*p* ≤ 0.01). The large variability over time in the activity or content of the plant constituents explains why the main effect of TiO_2_-NPs application itself was not large. We can state that TiO_2_-NPs affected the malondialdehyde content (*p* ≤ 0.01) and glutathione, L-ascorbic acid, and titanium content (*p* ≤ 0.001). The insignificant differences for the remaining parameters did not mean that the applied NPs had no effect on the plant parameters but made it necessary to look more closely at the interaction effects.

## 4. Conclusions

Based on our results, we conclude that TiO_2_-NPs influence the antioxidant status in oakleaf lettuce, but the greatest changes in the investigated components of antioxidant mechanisms were observed usually from 4 to 7 days after NPs treatment. This indicates that the used concentration of TiO_2_-NPs (0.75%) turned out to be sufficient to cause oxidative stress but was not high enough to induce severe stress and to seriously damage the plants. It can be assumed that the plants were able to restore the balance between ROS generation and ROS scavenging, though not necessarily entirely; however, the levels of some antioxidants (e.g., glutathione) remained significantly higher in the treated plants throughout the whole period of the experiment. The reduction of Ti toxicity could be due to the GSH properties as metal chelator. A positive effect of TiO_2_-NPs on plant biomass was noted. However, it was observed only at 7 days after NPs treatment.

## Figures and Tables

**Table 1:** ANOVA results for estimated parameters of oakleaf lettuce seedlings treated with TiO_2_-NPs as influenced by nanoparticles (NPs), sampling time (tn), and the interaction NPs × tn.

Parameter	Nanoparticles (NPs)	Sampling Time (tn)	NPs × tn
Malondialdehyde	**	**	***
Total antioxidant capacity	ns	***	***
Glutathione	***	**	***
L-ascorbic acid	***	***	***
Total phenolics	ns	***	***
Ascorbate peroxidase	ns	***	***
Guaiacol peroxidase	ns	***	***
Fresh weight	ns	***	***
Dry weight	ns	***	***
Titanium	***	***	***

Level of significance: ** *p* ≤ 0.01; *** *p* ≤ 0.001; ns—not significant.

## Data Availability

Most data supporting the results are included in the article. The datasets used and/or analyzed during the current study are available from the corresponding author on reasonable request.
